# Clinical End-Points Associated with *Mycobacterium tuberculosis* and Lung Cancer: Implications into Host-Pathogen Interaction and Coevolution

**DOI:** 10.1155/2015/827829

**Published:** 2015-10-25

**Authors:** Yansheng Tian, Tong Hao, Bin Cao, Wei Zhang, Yan Ma, Qiang Lin, Xiaomin Li

**Affiliations:** ^1^Central Laboratory, North China Oilfield Hospital of Hebei Medical University, Renqiu, Hebei 062552, China; ^2^Department of Pathology, North China Oilfield Hospital of Hebei Medical University, Renqiu, Hebei 062552, China; ^3^Department of Pharmacy, North China Oilfield Hospital of Hebei Medical University, Renqiu, Hebei 062552, China; ^4^Oncology Department, North China Oilfield Hospital of Hebei Medical University, Renqiu, Hebei 062552, China

## Abstract

There is a recent emerging theory that suggests a cross-link between pathogens and cancer. In this context, we examined the association between the *Mycobacterium tuberculosis* (MTB) with its L-forms (MTB-L) and lung cancer. In the present study, we have optimized and applied a highly sensitive assay to detect the presence of MTB and MTB-L in 187 lung cancer samples and 39 samples of other cancer origins. By carefully controlling confounding factors, we have found that 62% of the lung cancer samples are MTB-L positive, while only 5.1% of the other cancer samples are MTB-L positive. Through generalized linear models and random forest models, we have further identified a set of clinical end-points that are strongly associated with MTB-L presence. Our finding provides the basis for future studies to investigate the underlying mechanism linking MTB-L infection to lung cancer development.

## 1. Introduction

Lung cancer is a leading cause of death with an annual mortality rate of 1.59 million people, accounting for 19.3% of all cancer mortality worldwide [1]. Of notable concern in China, lung cancer mortality rate has continuously risen for the past three decades at an alarming annual rate of 464%. This has now replaced liver cancer as the leading cause of death from malignant tumors [2]. Therefore, understanding the causes of lung cancer is of great importance.

It has been speculated that* Mycobacterium tuberculosis* (MTB), primarily as a pathogen of the mammalian respiratory system, is closely linked to the occurrence of lung cancer [3]. Approximately one-third of the people in the world have been infected by MTB [4], with 1% of the world population becoming newly infected each year [5]. Of particular interest, 90–95% of MTB carriers have no symptoms [6].* Mycobacterium tuberculosis* L forms (MTB-L) are a wall-defective and pleomorphic form of MTB and have many characteristics different from MTB's original vegetative forms [7]. While investigating the incidence of lung cancer, researchers noticed that MTB-L, which have no cell wall, may act like some carcinogenic viruses that can induce cancer by DNA integration [7].

Generally, MTB-L have low pathogenicity and activity. Therefore, MTB-L have often gone undiagnosed and have not been identified as the cause of disease [7]. For example, the clinical presentations of MTB-L may be misclassified as “chronic lymphadenitis of unknown origin,” because inflammation may be the only detectable sign. This often happens because of other viruses or mycoplasma infections [7]. Consequently, to avoid failing to detect or misdiagnosing MTB-L, pathologists have adopted three sensitive methods: (1) acid-fast, (2) immunohistochemical staining, and (3) isolation. It has been reported that, among acid-fast staining methods, Intensified Kinyoun's (IK) method has higher sensitivity than the Ziehl-Neelsen (ZN) method [7] and has emerged as the main acid-fast staining method. However, the sensitivity of the IK method to detect MTB is still improvable. As a result, probe-based methods with increasing performance are being rapidly developed for MTB (including MTB-L) detection. In this paper, in order to improve the sensitivity of MTB and MTB-L detection, probes targeting gene* mpb64* are used in* in situ* hybridization. Gene* mpb64* is in the MTB genome, and its MPB64 protein is identified from MTB strain H37Rv [8, 9]. Although MTB-L cannot grow well in media for routine examination of tuberculosis, media culture remains the standard way to detect and isolate the infection. However, 92-3TB-L media were reported to be suitable for the growth of both MTB vegetative forms and L-forms [7]. In our lab, we developed a standardized protocol of using 92-3TB and 92-3TB-L media to maximize the isolation rate of L forms [10, 11].

Currently, there have been limited investigations on the relationship between lung cancer and MTB-L. Our lab is a key pioneer in this field and published two primary studies. In these two studies, we showed that MTB-L exist in lung cancer patients as the primary type of MTB. Unfortunately, we were unable to explain any of the tumorigenic mechanisms yet [10, 11].

In this study, we examined 187 lung cancer tissues for MTB/MTB-L presence by combining IK acid-fast staining and* in situ* hybridization using* mpb64* probes. We compared the rate of MTB/MTB-L positive cases in lung cancer with the rate among other cancer types. By computational methods, we were able to extract key clinical features that can characterize a subpopulation of lung cancer samples with higher prevalence of MTB/MTB-L.

## 2. Materials and Methods

### 2.1. Samples

Table 1 was the summary of clinical characteristics of all samples used in this study. There were three main groups of samples: (1) lung cancer samples, which were used to study the association between MTB/MTB-L and lung cancer; (2) tuberculosis group without cancer (TB, non-lung-cancer), which was used as the positive control to estimate the sensitivity of the MTB/MTB-L detection assay; and (3) a group of samples without TB (non-TB, non-lung cancer), including hepatic, gastric, and breast cancers, was used as the negative control to estimate the specificity for the MTB/MTB-L detection assay.

The lung cancer samples were from 187 patients who were diagnosed with lung cancer at the North China Oilfield Hospital of Hebei Medical University. Here, they had surgery to remove the cancer from the lung between 2003 and 2012. A consensus cancer diagnosis was reached by pathology. For 131 out of 187 patients, complete pathological records were available. For 47 out of 131 patients, fresh surgery tissues were available. Within the 19 patients with history of TB, sixteen patients had ipsilateral tuberculosis and cancer, while another three had contralateral disease.

For the TB, non-lung-cancer group, samples were from 31 patients who had surgery from the North China Oilfield Hospital of Hebei Medical University between 2004 and 2012 (14 out of 31 patients had fresh surgery tissues). Among the 31 patients, 19 had tuberculosis and 12 had tuberculous lymphadenitis.

For the non-TB, non-lung-cancer control group, samples were from 39 hospitalized surgical patients in the North China Oilfield Hospital of Hebei Medical University between 2004 and 2012. Each patient went through a chest X-ray, CT, and OT tests that excluded the possibility of patients for having tuberculosis or lung cancer. 17 out of 39 patients had fresh surgery tissues. Among the 39 patients, 6 had hepatic carcinoma, 14 had gastric cancer, and 19 had breast cancer.

The research protocol was approved by ethic committees of the North China Oilfield Hospital of Hebei Medical University where the samples were collected and all patients provided informed consent. All specimens were handled and made anonymous according to the ethical and legal guidelines.

### 2.2. Reagents

Targeted fragments My250-224 and My310-283 were chosen from the* mpb64* gene (X75361, 826 bp) from the standard strain* Mycobacterium tuberculosis* H37R. The probes for the two fragments were made and biotin-labeled by Tianjin Haoyang Biotechnology Company (Tianjin, China.) The probe sequences were as follows:

1. 5′-CGGTATCGGTGCCTTTCAACTCCTCGC-3′,
2. 5′-GGGCAGGCTGATGTTGATGTTGTAGGC-3′.

The probes can be used separately or mixed together. In order to increase the MTB detection rate, these two probes were mixed for this experiment. The* in situ* hybridization kit was purchased from Tianjin Haoyang Biotechnology Company. Other reagents such as 92-3TB, 92-3TBL, PNB-MTB and PNB-MTB-L, TCH-MTB and TCH-MTB-L, and other liquid media were prepared by our lab.

92-3TBL liquid media were made by first dissolving 2 g of glucose, 5 g of amino succinamic acid, 2.5 g of monopotassium phosphate, 1 g of monometallic sodium orthophosphate, 2.5 g of sodium citrate, 0.5 g of magnesium sulfate, 4 g of gelatin, 30 g of sodium chloride, and 20 mL of glycerol into distilled water to a final volume of 900 mL. (All materials were purchased from Beijing Chemical Works, Beijing, China.) 4% NaOH was added to the solution to adjust its pH to 7.0. 1 mL of 1% malachite green (Ziyi Reagent Company, Shanghai, China) that was added to the solution, which was then split into vaccine bottles with 90 mL in each bottle. Bottles were placed under eight pounds of pressure for 15 minutes. After cooling to room temperature, 10 mL of sterile blood plasma was added into each bottle. (Fresh blood plasma was kept under 56 degrees Celsius for one hour followed by 4 degrees Celsius overnight. Blood plasma was brought up the next day by centrifuging at 1500 ×g for 30 minutes.) Finally, aseptic technique was used to split the mixture into small test tubes with two to three mL in each tube. Samples were cultured in an incubator for 48 hours to ensure no bacterial contamination.

92-3TB liquid media was made following the same procedure as that for 92-3TBL liquid media, with the exception that no sodium chloride was added.

p-Nitrobenzoic acid- (PNB-) MTB or MTBL liquid media were made by adding PNB (Shanghai Chemical Works, Shanghai, China) into 92-3TB or 92-3TBL liquid media to make the final PNB concentration to 0.25 mg/mL.

2-Thiophenecarboxylic acid hydrazide- (TCH-) MTB or MTBL liquid media were made by adding TCH (Shanghai Chemical Works, Shanghai, China) into 92-3TB or 92-3TBL liquid media to make the final TCH concentration to 0.25 mg/mL.

### 2.3. Methods

Slide preparation: tissue was harvested and fixed immediately with 4% paraformaldehyde (Beijing CellChip Biotechnology Co., Ltd., Beijing, China) and 0.1 M PBS (PH 7.0 to 7.6 in 1/1000 DEPC (Sigma-Aldrich, St. Louis, MO, USA.)), followed by dehydration, paraffin embedding, and sectioning of a series of 6 slides with a thickness ranging from 4 to 6 *μ*m.

Intensified Kinyoun's (IK) acid-fast staining to detect MTB-L: as used here, this method has previously been described in the literature [10]. Briefly, 3 g of Schiff stain (Ziyi Reagent Company, Shanghai, China) was dissolved in 20 mL of 95% ethanol (Ziyi Reagent Company, Shanghai, China). 8 mL of phenol (Beijing Chemical Works, Beijing, China) was added into 80 mL of distilled water. The final solution was filtered for storage. Before each use, 0.1 mL of Tween 80 (Beijing Chemical Works, Beijing, China) was added into the mixture of the previous two solutions. Decolorant was made by dissolving 0.5% muriatic acid in ethanol solution, followed by adding 0.5 mL of concentrated hydrochloric acid (Beijing Chemical Works, Beijing, China) into 100 mL of 95% ethanol (Ziyi Reagent Company, Shanghai, China). After staining, 0.1 g of methylene blue (Ziyi Reagent Company, Shanghai, China) was dissolved in 100 mL of 0.2% acetic acid (Beijing Chemical Works, Beijing, China). Detection of MTB-L was based on the frequency of finding a stained cell in a field of a microscope. If 0 out of 300 fields of a microscope was found, it was marked as MTB-L negative (−); if 1 to 2 out of 300 fields were found, it was marked as MTB-L inconclusive (±); otherwise, if a range of 3 or more out of 300 fields were found, it was marked as MTB-L positive (+).

The Ziehl-Neelsen staining to detect MTB-bacteria type: the slides were dewaxed and went through the following series of ethanol dehydration (Ziyi Reagent Company, Shanghai, China). First, carbol fuchsin solution was made by dissolving 4 g of Schiff stain (Ziyi Reagent Company, Shanghai, China) in 100 mL of 95% ethanol (Ziyi Reagent Company, Shanghai, China) to make 100 mL of saturated solution. Second, 10 mL of saturated solution was mixed with 90 mL of 5% phenol (Beijing Chemical Works, Beijing, China). Third, 3% muriatic acid ethanol solution was made by adding 3 mL of concentrated hydrochloric acid (Beijing Chemical Works, Beijing, China) into 97 mL of 95% ethanol (Ziyi Reagent Company, Shanghai, China). Lastly, Loffler's alkaline methylene blue solution was made by dissolving 2 g of methylene blue (Ziyi Reagent Company, Shanghai, China) into 100 mL of 95% ethanol (Ziyi Reagent Company, Shanghai, China) to make 100 mL of saturated solution. The final solution was made by taking 30 mL of saturated solution and mixing it with 100 mL of distilled water and 0.1 mL of 10% caustic potash (Beijing Chemical Works, Beijing, China). These methods strictly followed TB Diagnostic Bacteriology Testing Protocols developed by the China Tuberculosis Association.

*In situ* hybridization to detect* mpb64* gene expression: following the procedure from the kit manual, the probe was hybridized to a lung cancer tissue slice and used as the positive control, while a lung cancer tissue without probe hybridization was used as the negative control. The detection method was NBT/BCIP method. If the hybridized cancer cell nucleus showed brown particles, it was deemed* mpb64* positive. To determine the* mpb64* expression status per slice, five high magnification views (400x) from each slice were selected at random. A* mpb64*-positive-cancer-cell rate for each view was determined by counting the percentage of* mpb64*-positive cancer cells in all cancer cells under each view. The final positive rate for each slice was then calculated as an average of the rates from the five views. The overall sample's* mpb64* expression was deemed positive if the per-slice positive rate is greater than or equal to 5%; otherwise, it was labeled as* mpb64* negative.

Use of bacterial culture to detect MTB and MTB-L: part of the tissue from each resecting surgery was placed onto a sterile plate, cut by sterile scissors, and ground. Each half gram of ground tissue was then inoculated into 92-3TB and 92-3TBL liquid media, mixed, and incubated at 37 degrees Celsius for one to three weeks. The sample was observed once each week by taking the precipitate onto slides for the IK acid-fast staining and the Ziehl-Neelsen staining before microscopy observation. Bacteria samples that were grown on 92-3TB liquid media were transferred to PNB-MTB and TCH-MTB liquid media to identify the MTB type. Bacteria samples that were grown on 92-3TBL liquid media were passed to the same media (92-3TBL) from generation to generation. Each generation was cultured for one week until the fifth generation. Meanwhile, samples were inoculated onto PNB-MTL-L and TCH-MTB-L liquid media. The identification method for MTB-L was the same as for MTB above.

### 2.4. Classification Model of MTB-L with Clinical End-Points

#### 2.4.1. Generalized Linear Model (GLM)

The response of the data was calculated with following equation:

(1) $$\begin{array}{c} Response=IK*MPB64. \end{array}$$

Samples with missing values were filtered out. The final dataset contained seven covariates and 99 samples (Table 2), 69 of which had a positive response.

All covariates were put into an initial model. Next stepwise Akaike's Information Criterion (AIC) was applied to pick the strongest predictive features as well as to avoid overfitting. This procedure was repeated 1000 times, and the frequency of each variable appearing in the best model was used to determine the “important” features. Due to the unbalanced group sizes, in each iteration, we utilized the bootstrap resampling technique to generate 100 positive samples and 100 negative samples as the training data. Pseudo *r* square was calculated for investigating goodness-of-fit. The training data was predicted by the model, and AUC was calculated as a measure of model accuracy. All statistics analyses were done with the R programming language [12].

#### 2.4.2. Random Forest Model

Random forest is an ensemble of classification trees that are calculated on random subsets of the data, using a subset of randomly restricted and selected predictors for each split in each classification tree. In this study, we used the “party” R package [13] to perform random forest modeling. Again as the samples were not evenly distributed, we utilized bootstrap resampling to create a balanced dataset with 100 positive samples and 100 negative samples, which was then used for random forest modeling. We repeated this procedure five times to get average accuracy estimation. The “party” R package also provides a permutation accuracy variable importance measure. Variables were considered informative and important if their variable importance values were above the absolute value of the lowest negative-scoring variable. The rationale for this rule of thumb is that the importance of irrelevant variables varies randomly around zero. In order to model the random feature, we made two features by scrambling the response vector and then running random forest with the two random features.

## 3. Results

### 3.1. Development of a Reliable Detection Assay for MTB/MTB-L

Originally, we sought to establish a reliable assay for detecting MTB-L by assessing and optimizing existing standard assays. The Ziehl-Neelsen (ZN) staining had traditionally been used as a standard method for detecting MTB (all forms) in TB samples. Recently, reports have leveraged the emergence of Intensified Kinyoun's (IK) acid-fast staining technique to detect MTB in general, and more specifically the L forms [10, 14].

**Figure 1:**
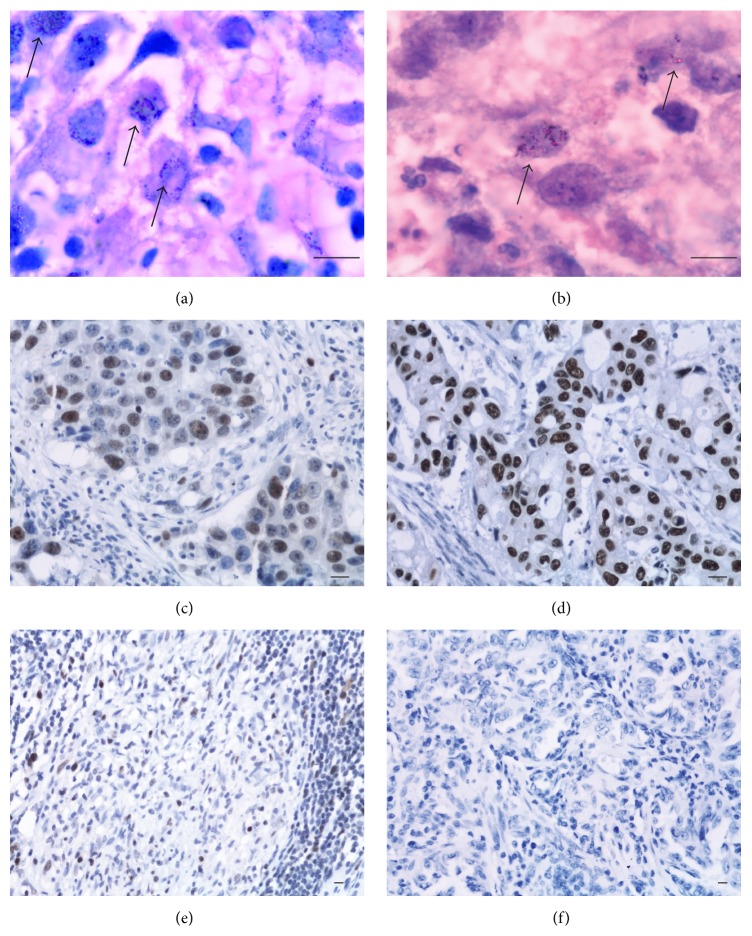
Sample histology images. The scale bar equals 10 *μ*m in each figure. (a and b) The IK staining figures with 1000 times zoomed in. (a) Showing MTB-L in the nucleus of squamous cell carcinoma (see black arrows). (b) Showing MTB-L in the nucleus of lung adenocarcinoma (see black arrows). (c and d) 400 times zoomed in* in situ* hybridization figures. (c) Showing* mpb64* positive expression in the nucleus of squamous cell carcinoma. (d) Showing* mpb64* positive expression in the nucleus of lung adenocarcinoma. (e and f) 200 times zoomed in* in situ* hybridization figures. (e) Positive control showing* mpb64* in tuberculosis. (f) Negative control where no* mpb64* probes are added in lung adenocarcinoma.

**Figure 2:**
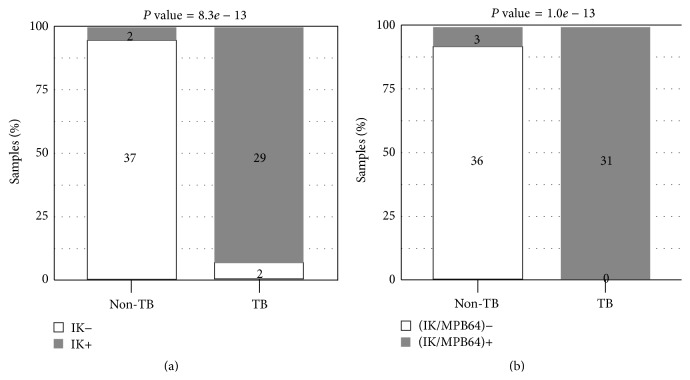
Assessment and optimization of MTB/MTB-L detection assay. 31 TB, non-lung-cancer samples and 39 non-TB, non-lung-cancer samples were used as the test set. As the cause of TB, MTB-L was expected to be present in the TB, non-lung-cancer but absent in the non-TB, non-lung-cancer samples. (a) Detection of MTB-L by the IK method alone. (b) Detection of MTB/MTB-L by the IK method and the* mpb64* marker.

In the current report, we first assessed our ability to detect MTB-L by the IK method. Discriminatory detection of MTB-L by this method has a significant histology component (see Methods) and depends on the experience of the histologists (Figure 1). A sample set containing 31 TB, non-lung-cancer samples and 39 non-TB, non-lung-cancer samples was tested. Non-lung-cancer samples were used to eliminate lung cancer as a potential factor affecting the detection of MTB-L in tissues. As shown in Figure 2(a), 29/31 (93.5%) TB, non-lung-cancer samples were correctly identified as IK-positive (for MTB-L), while 37/39 (94.9%) non-TB, non-lung cancer samples were correctly identified as IK-negatives. For reference, the ZN staining was also performed on a subset of these samples for confirmatory purposes with 100% agreement (results not shown). To further confirm the diagnosis of MTB-L, 31 TB samples were used to isolate and culture bacteria under the appropriate selection media conditions for MTB-L [11]. All 31 TB samples were confirmed to have MTB-L by the culture methods. Conversely, culture results from 16 non-TB samples that were IK-negative had a 100% confirmation rate as being negative for MTB-L.

We next explored novel ways to improve positive detections from TB, non-lung-cancer samples. We reasoned that since the leftover samples contained MTB/MTB-L in a way that was hard to be stained by the IK method, we could make use of genomic information from MTB to improve sensitivity. Meanwhile, we hypothesized that these hard-to-stain MTB samples might have unknown forms of MTB, which had deviations from the textbook characteristics and were worth further study. As a result, we complemented the IK method with a genetic component by testing for the presence of the* mpb64* gene in the cell nucleus, a marker for MTB [11]. The combination of the IK method and the* mpb64* marker improved the true positives rate in TB samples for MTB/MTB-L from 93.5% to 100% while only slightly decreasing the true negatives rates in non-TB samples from 94.9% to 91.7% (Figure 2(b)). Based on these results, we adopted the combination of the IK method and the* mpb64* marker as the extended criteria for classifying MTB/MTB-L presence for the remaining studies in this report, while the IK method alone was the strict criteria for classifying MTB-L presence.

### 3.2. In Patients with No TB History, MTB-L Is More Prevalent in Lung Cancer Samples Compared to Other Cancer Types

**Figure 3:**
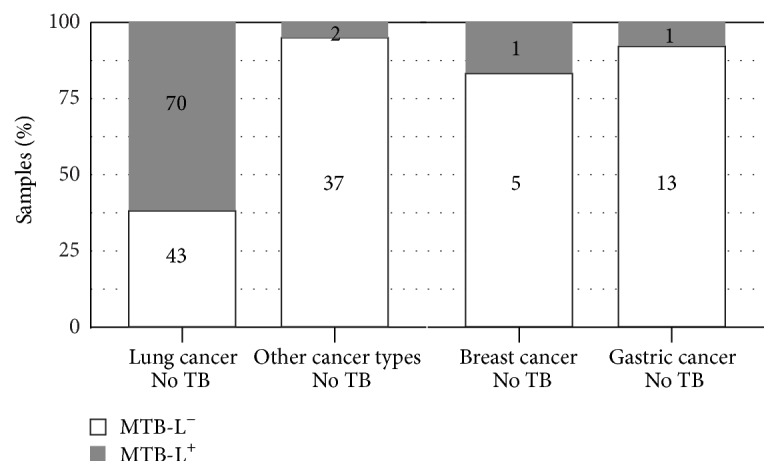
Higher presence of MTB-L in the lung cancer samples compared to samples of other cancer types by the IK method alone.

**Figure 4:**
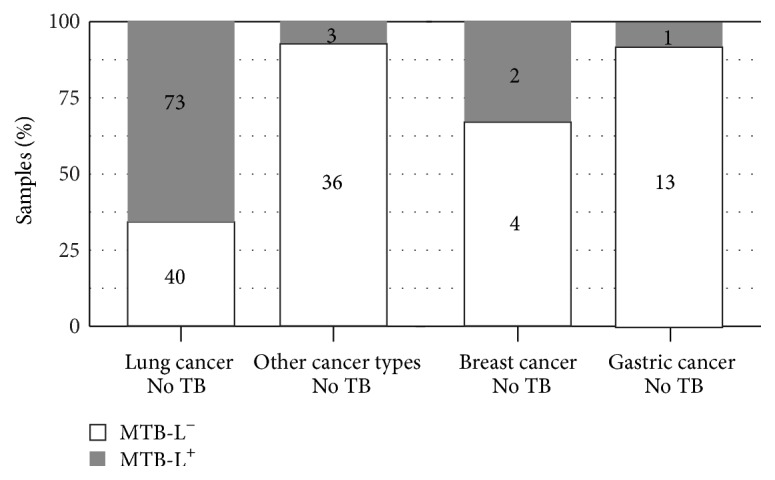
Higher presence of MTB/MTB-L in the lung cancer samples compared to samples of other cancer types by both the IK method and the* mpb64* marker.

A total of 187 lung cancer samples were collected for the present study. Of these samples, 19 patients had a documented prior medical history of TB and 113 had no reported TB history, while the remaining 55 patients were missing this information. We selected the 113 samples with no reported TB history to compare with 39 samples of other cancer types without prior TB medical history. As shown in Figure 3, MTB-L was detected from 70 of the 113 lung cancer samples (61.9%), a statistically significant (*P* value = 2.2*e* − 16) contrast with the samples from other cancer types (2 of 39, 5.1%). Among other cancer types, there were one out of 14 gastric cancer samples (7.1%) and one out of six breast cancer samples (16.7%) detected as MTB-L positive, which was still a statistically significant contrast. To increase sensitivity (Figure 4), MTB/MTB-L was detected from 73 of the 113 lung cancer samples (64.6%), which was still significantly (*P* value = 2.6*e* − 15) higher than 3 of 39 samples from other cancer types (7.7%). Among other cancer types, there were one out of 14 gastric cancer samples (7.1%) and two out of six breast cancer samples (33.3%) detected as MTB-L positive.

To investigate the relation between MTB and breast cancer, we conducted a breast cancer study. Out of 115 breast cancer patients, we found that 53 samples (46.1%) were MTB-L positive, which is statistically significantly (*P* value = 0.016) lower than 61.9% in lung cancer. For MTB/MTB-L, 60 out or 115 samples (52.2%) were positive, which was still lower than 64.6% in lung cancer (*P* value = 0.057) [15].

### 3.3. The Presence of MTB-L Is Associated with a Specific Subset of the Lung Cancer Population

We hypothesized that, within the lung cancer patients, the MTB-L positive population represents a homogenous subset that shares a common set of measurable clinical end-points. No apparent connection, however, could be found between the presence of MTB-L and individual clinical end-points in isolation (Table 3).

We next applied a generalized linear regression model as a representative of linear models to examine potential combinatorial effects of all clinical end-points. The final model identified category and lymph node metastasis as having the strongest combined effects (Table 4). The strong predictive power of the model (AUC = 0.70) elucidated the necessity of examining the combinatorial effect of the four clinical features. The suboptimal *r*-squared value (0.1), however, indicated that a linear model alone is likely insufficient, especially without explicit inclusion of critical interaction terms.

**Figure 5:**
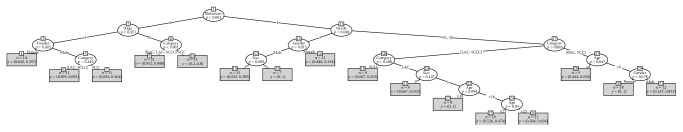
One example of decision trees from the random forest model. In the annotation: metastasis means lymph node metastasis; grade means pathology grade; stage means clinical stage; size means tumor size.

We then removed the assumption that the association is strictly linear and derived a random forest model as a representative of nonlinear models to reexamine the association. Again random forest model was run on a balanced dataset through bootstrapping. We repeated the procedure five times and the average AUC was 0.85 (0.850, 0.845, 0.850, 0.885, and 0.830). We used a standard R package to run random forest model, which should be replicable.  Figure 5 was one example of the tree structure. The feature importance ranking agreed with our linear model and selected the most dominant features (category, pathology grade, lymph node metastasis, and clinical stage). Age was significant as well. By comparing the linear and nonlinear models, we found that nonlinear models performed better. This was consistent with our hypothesis that features are nonlinearly associated with each other in terms of predicting outcomes.

## 4. Discussion

The relatively high rates of lung cancer and TB cases in China have recently sparked new theories that there might be cross-associations between the two diseases. Recent reports of colocalization of MTB/MTB-L in tumor samples, although limited in sample size and coincidental in nature, underline the need for thorough confirmation studies. Towards this goal, we set up the present study with three key components in mind: (1) accurate detection assay; (2) large sample size; and (3) full medical history and clinical diagnosis to control for confounding factors as well as linkage to clinical end-points.

Acid-fast staining methods are the traditional way for MTB and MTB-L detection, and isolation is the gold standard for validation. However, we noticed that staining methods failed to detect MTB or MTB-L in some MTB-L positive samples that were validated by isolation. We hypothesized that there might be a group of MTB which is hard to be stained because of unknown characteristics. In this situation, because this group should still contain the main components of MTB, we could make use of an important MTB protein coding gene (*mpb64*) for detection. In this study, we proved that the IK method is better than the ZN method, but the* mpb64 in situ* hybridization method can further increase sensitivity. By combining the IK method and the* mpb64 in situ* hybridization method, we could find out samples that had MTB but in a hard-to-stain form, which is worth future study.

We performed MTB/MTB-L detection on all 187 samples isolated from lung cancer patients but decided to concentrate on the 113 samples that had no history of TB. It was critical to obtain samples from other cancer types that were also free of prior TB history. By leveraging the available medical records, we were able to remove TB history as a potential confounding factor—an important step in the subsequent interpretation of MTB-L presence. Central to our key findings, 61.9% of the lung cancer samples were found to be MTB-L positive, a statistically significant higher percentage than that of the other cancer samples (5.1%) or that of gastric cancer sample (7.1%); see Figure 3. For breast cancer, we had only 6 samples, which were not enough to make statistical conclusion. The two positive samples were both validated to have MTB-L by isolation method. This fact implied that MTB/MTB-L might also have association with breast cancer, which was confirmed by our breast cancer study [15].

This finding suggested that the presence of MTB-L might be strongly associated with lung cancer, but the causal relationship was underdetermined. Because MTB-L are wall-defective and pleomorphic, they have the potential to act like carcinogenic viruses. They can get into human alveolar epithelial cells, remain dormant, and induce cancer by DNA integration, because DNA integration can potentially activate oncogenes and/or repress tumor suppressor genes. In mouse model, researchers reported that MTB-L infected mouse had significant higher chance to develop neoplasia than the control group [16, 17]. There are well-known viruses that can lead to cancer, for example, human papilloma viruses, hepatitis B and C viruses, and so forth. However, even for these viruses, host-pathogen adaptation and biological mechanism of tumor causality are still unclear. Similarly, the interaction between host and MTB-L is not fully understood yet.

While the biological mechanism of causality is still unclear, we acknowledged that preclinical experiments beyond the scope of the present report were needed to fully investigate the question of causality. Nevertheless, we reasoned that a causal relationship would also reflect an association with clinical disease severity.

Towards this goal, we focused on finding end-point differences between MTB-L positive and negative lung cancer samples. None of the individual end-points in isolation was sufficient to distinguish between the positive and negative samples. However, upon applying computational classification algorithms, we were able to identify a set of clinical end-points that could be used to stratify between the samples. To our knowledge, this was the first report linking clinical end-points to the presence and absence of MTB-L. As our rule-based model indicated, MTB-L was especially prevalent in patients with non-lymph-node-metastatic small-cell lung and squamous cell carcinoma. Our findings should lead to further studies focusing on this subpopulation, as well as making a strong case for the advantage of utilizing primary cells from this subpopulation as an experimental model system.

## Key Points

 A reliable detection assay for MTB/MTB-L was developed.
 MTB-L is more prevalent in lung cancer samples compared to other cancer types.
 A set of clinical end-points, which are strongly associated with MTB-L presence, was identified.

## Figures and Tables

**Table 1:** Patient characteristics.

Characteristic	Values	Lung cancer	TB, non-lung-cancer	Non-TB, non-lung-cancer
Eligible patients	Total number	187	31	39

Category WHO (lung cancer histological classification) of 2004	Squamous cell carcinoma (SCC)	63	NA	NA
	Lung adenocarcinoma (LAC)	77	NA	NA
	Bronchioloalveolar carcinoma (BAC)	27	NA	NA
	Small cell lung carcinoma (SCLC)	20	NA	NA

Clinical stage (UCC 1997)	I, II	61	NA	NA
	III, IV	70	NA	NA

Tumor size	≤3 cm	56	NA	NA
	>3 cm	75	NA	NA

Lymph node metastasis	Yes	89	NA	NA
	No	42	NA	NA

History of TB	Yes	19	31	NA
	No	112	0	NA

Gender	Male	80	26	14
	Female	107	5	27

Pathology grade	I	22	NA	NA
	II	60	NA	NA
	III	63	NA	NA

Age		59.3 (mean), 10.3 (sd)	53.4 (mean), 9.1 (sd)	49.9 (mean), 7.6 (sd)

**Table 2:** IK *∗* MPB64_TCN_ISH as response. 99 samples, positive : negative = 69 : 30.

Clinical endpoints	*P* value	Values	MTBL positive	MTBL negative
Category	0.0832	Squamous cell carcinoma (SCC)	29	6
		Lung adenocarcinoma (LAC)	28	13
		Bronchioloalveolar carcinoma (BAC)	8	6
		Small cell lung carcinoma (SCLC)	4	5

Clinical stage	0.4328	I, II	27	15
		III, IV	42	15

Tumor size	0.9732	≤3 cm	28	13
		>3 cm	41	17

Lymph node metastasis	0.1409	Yes	49	16
		No	20	14

Gender	0.3929	Male	31	10
		Female	38	20

Pathology grade	0.4674	I	13	6
		II	34	11
		III	22	13

Age	0.9579		59.86 (mean), 9.53 (sd)	59.73 (mean), 10.89 (sd)

**Table 3:** IK as response. 112 samples, positive : negative = 77 : 35.

Clinical endpoints	*P* value	Values	MTBL positive	MTBL negative
Category	0.2246	Squamous cell carcinoma (SCC)	30	8
		Lung adenocarcinoma (LAC)	32	18
		Bronchioloalveolar carcinoma (BAC)	8	7
		Small cell lung carcinoma (SCLC)	7	2

Clinical stage	0.9386	I, II	33	16
		III, IV	44	19

Tumor size	1	≤3 cm	33	15
		>3 cm	44	20

Lymph node metastasis	0.4841	Yes	53	21
		No	24	14

Gender	0.1879	Male	36	11
		Female	41	24

Pathology grade	0.6250	I	14	7
		II	36	13
		III	27	15

Age	0.8946		60.00 (mean), 9.86 (sd)	60.26 (mean), 9.31 (sd)

**Table 4:** Feature frequency table of generalized linear model.

Covariable name	Frequency (%)
Category	98.6
Gender	39.6
Pathology grade	62.1
Lymph node metastasis	93.9
Tumor size	65.8
Clinical stage	54.3
